# Percutaneous Retrograde Trans-Gluteal Embolization of Type 2 Endoleak Causing Iliac Aneurysm Enlargement after Endovascular Repair: Case Report and Literature Review

**DOI:** 10.3390/jcm13102909

**Published:** 2024-05-15

**Authors:** Andrea Esposito, Rocco Pasqua, Danilo Menna, Antonio Nicola Giordano, Giulio Illuminati, Vito D’Andrea

**Affiliations:** 1 Vascular and Endovascular Surgery Division, Cardiovascular Department, San Carlo Hospital, 85100 Potenza, Italy; miandra@libero.it (A.E.); dmenna@hotmail.it (D.M.);; 2 Department of Surgery, Sapienza University of Rome, 00185 Rome, Italy; giulio.illuminati@uniroma1.it (G.I.); vito.dandrea@uniroma1.it (V.D.)

**Keywords:** endoleak, internal iliac artery, endovascular aortic repair, endovascular technique, superior gluteal artery, embolization

## Abstract

Late type II endoleaks (T2ELs) arising from the internal iliac artery (IIA) may present during follow-up after endovascular aortic repair (EVAR) of aortoiliac aneurysm and may warrant embolization if enlargement of the aneurysmal sac is demonstrated. When coverage of the IIA ostium has been made due to extensive iliac disease, access options can be challenging. Different treatment options have been reported over recent years, and a careful selection of the best one must be made based on the characteristics of each case. The present study reports a simple and reproducible sheathless percutaneous superior gluteal artery (SGA) access and provides a discussion based on a review of the existing literature on this topic.

## 1. Introduction

Endovascular aneurysm repair (EVAR) is the treatment of choice for infrarenal aortic aneurysms with fit anatomy. It showed significantly higher short-term survival rates compared with open surgery, but long-term survival rates are equivalent, and endovascular results are impaired by higher rates of aneurysm-related reinterventions. The principle of endovascular treatment of aneurysms is the exclusion of the diseased arterial segment from circulation so as to no longer expose the segment itself to arterial pressure. This principle is achieved through the placement of an endograft, which requires, as a prerequisite for its correct functioning, the presence of a healthy artery at its proximal and distal edges. Any flow into the aneurysmatic sac outside the graft after EVAR represents an endoleak that is an important cause of the recurrence of disease and reintervention. Endoleaks are classified as primary when they appear immediately after the endovascular repair or secondary when they appear during the follow-up after prior imaging that showed no irregularities. Type I endoleaks are classified as direct flow into the residual aneurismal sac through proximal or distal sealing zones due to poor adherence between the endograft and the arterial wall. Because of their high risk of rupture, they need prompt management. Type II endoleaks are classified as retrograde flow from collaterals arising from the excluded arterial segment. Type III endoleaks result from stent graft component maldeployment, migration, or fabric tear. Leakages through the stent graft material porosity are defined as Type IV endoleaks. Type V endoleaks, also named endotension, are defined by sac expansion without any visible endoleak. The focus of this study is on secondary endoleaks, also known as late type II endoleaks (L2ELs). They can occur after EVAR, with a prevalence at 1-year follow-up of up to 16% [1]. Their clinical significance remains controversial, but surveillance is necessary, and treatment is suggested when the aneurismal sac increases to prevent rupture [2]. Their persistence, when associated with aneurismal sac expansion, represents the most common cause of aneurysm-related reinterventions. Both open and endovascular surgery techniques have been described in this setting, and the endovascular approach is nowadays the preferred option in patients with fit anatomy due to its shorter length of post-operative stay and lower morbidity compared with open surgery [3]. Many endovascular options exist, aiming to embolize the aneurysmal sac and feeding vessels. However, it may be difficult to reach the nidus of the endoleak by an anterograde route when a previous endovascular procedure has just been made. In particular, it may be difficult to reach the hypogastric artery with an anterograde route when coverage of the external iliac artery has been made due to extensive iliac disease. The aim of the present study is to report the case of a T2EL of the common iliac artery (CIA) retrograde embolization with a percutaneous sheathless superior gluteal artery (SGA) access after a previous plug embolization of the internal iliac artery (IIA) with external iliac artery (EIA) extension. A comprehensive literature review of different strategies and a critical analysis are also provided.

## 2. Case Presentation

**Figure 1:**
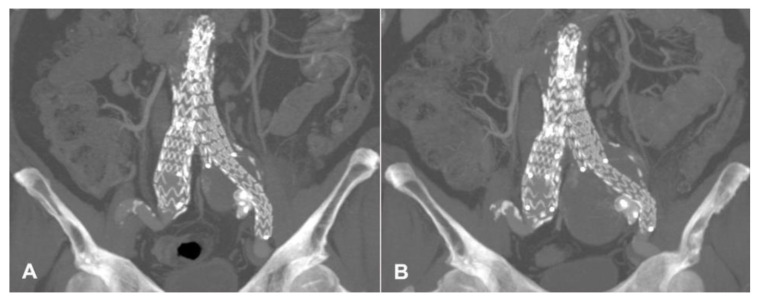
Enhanced computed tomography scan showing the left common iliac artery aneurysm after coil embolization and external iliac extension (**A**) and its enlargement with late contrast phase endoleak at 3-year follow-up (**B**).

**Figure 2:**
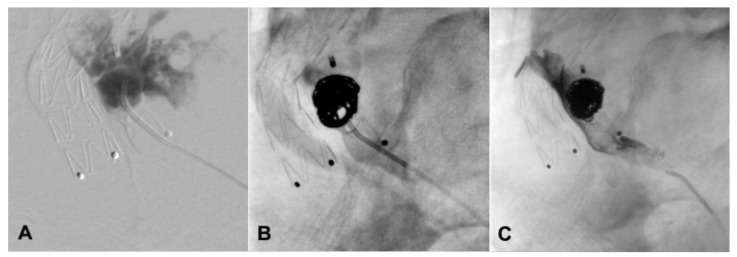
Intraoperative angiography through the left hypogastric artery showing the common iliac artery aneurism nidus of the endoleak (**A**), coil embolization with embolizing liquid agent (**B**), and completion angiography with no further nidus visualization (**C**).

**Figure 3:**
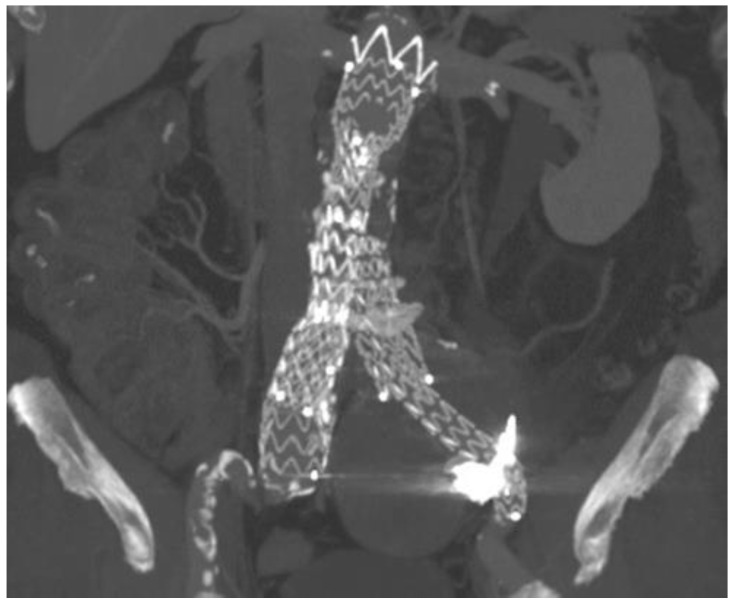
One-year enhanced CT scan showing left common iliac artery aneurysmatic sac stability and no endoleaks during the late contrast phase.

In 2010, a 63-year-old man underwent elective EVAR for a 65 mm asymptomatic infrarenal aortic aneurysm discovered at a computed tomography (CT) scan conducted as a completion of the aortic study due to the presence of an ascending aorta dilation. Other comorbidities included hypertension, hypercholesterolemia, mesangial glomerulonephritis, and seronegative polyarthritis. After an initial loss to follow-up, in 2018, he presented with a 50 mm left common iliac artery aneurysm, shown at an enhanced CT scan. Treatment consisted of iliac limb extension (Medtronic Endurant II, Minneapolis, MN, USA) into the external iliac artery and embolization of the main trunk of IIA with a 16 mm Amplatzer Vascular Plug (AGA Medical, Minneapolis, MN, USA). After 3 years, a CT scan demonstrated persistent enlargement of the left common iliac artery aneurysm, reaching 7 cm in diameter without clarifying its source (Figure 1). An angiography through right femoral access was planned. It showed a T2EL arising from the left IIA network with late enhancement of the left CCA aneurysm with no evidence of a clear collateral fueling the aneurismal sac and the correct position of the previous plug with no retrograde flow. After multidisciplinary discussion about treatment and access options, sac embolization through percutaneous SGA access was planned. The procedure was conducted in an angiographic suite under general anesthesia after systemic heparinization (5000 UI). At first, through a left brachial access, a 4Fr pigtail catheter (Cordis, Miami Lakes, FL, USA) was positioned in the right CIA to angiographically identify the contralateral SGA. Then, the patient was placed in a prone position. Under duplex ultrasound (DUS) and angiographical guidance, a direct puncture of the left SGA was performed using an 18-gauge, 100 mm long needle. A brief video of the diagnostic angiography and the sheatless SGA access are provided as Supplementary Materials. Over a 0.035-inch teflon-coated guidewire, a 4 Fr Ber catheter (Cordis Tempo, Miami Lakes, FL, USA) was advanced in a sheathless fashion to the main trunk of the left IIA. Then, it was pushed beyond the plug in a transealing fashion using a hydrophilic stiff guide (Terumo Glidewire, Tokyo, Japan), thus reaching the nidus of the leak. An intrasac angiography showed several small vessels directly feeding the aneurismal sac coming from the ipsilateral hypogastric network. A 2.7 Fr microcatheter (Rebar, Medtronic, Minneapolis, MN, USA) was placed in a coaxial fashion into the nidus, and 3 4 × 60 cm Ruby coil detachable coils (Penumbra, Alameda, CA, USA) were deployed to provide a scaffold. Then, 6 mL of a liquid embolic agent (Onyx 34, Medtronic, MN, USA) was used to fill the flow channel, as shown in Figure 2. Completion angiography demonstrated no further filling of the leak. Hemostasis of the puncture site was obtained by manual compression. In the postoperative period, no ischemic or hemorrhagic complications were observed, and the patient was discharged on the second postoperative day. A one-year follow-up CT scan demonstrated the stability of the left iliac aneurysm (Figure 3). Informed consent for publication was obtained by the participant.

## 3. Literature Review

### 3.1. Terms of Search and Inclusion Criteria

The preferred Reporting Items for Systematic Reviews and Meta-Analyses (PRISMA) statement guidelines were observed to conduct this review. Research on PubMed, Scopus, and the Cochrane Library for studies investigating the association between EVAR and the occurrence of type II endoleaks from hypogastric arteries and secondary reinterventions was performed. The first search was conducted on 1 June 2023. The following combination of keywords was used and connected through Boolean operators to maximize the sensitivity of the research: “embolization” AND “internal iliac artery” AND “superior gluteal artery” OR “endovascular aortic repair” AND “internal iliac artery” AND ”superior gluteal artery” OR “EVAR” AND “internal iliac artery” AND “superior gluteal artery”. Related articles and references were screened for suitable articles. The last database search was completed on 1 December 2023. Only articles in the English language containing specific information about the materials used for implantation were considered. Articles providing other arterial access techniques were collected to report a full-range of literature options and are presented in the Discussion section.

### 3.2. Data Extraction from Included Studies

Data from the included studies were extracted by two authors independently (R.P. and A.E.). The following data were collected: arterial access site, technique of access site, feeding vessel embolization, aneurismal sac embolization, embolic agents, technical success, hemostasis technique, and follow-up length. Technical success was defined by a second-level imaging examination after the target intervention demonstrating no late contrast-phase enhancement of residual aneurismal sac or sac dimension stability.

A database with all studies reporting a common/internal iliac artery aneurysm treatment utilizing a gluteal artery access was built. The database included nine studies published between 2014 and 2023, with a total of 10 patients included. No emergency treatments were included in the database.

### 3.3. Patients’ Characteristics and Technical Aspects

The average age of participants ranged between 56 and 86 years, and 90% of the patients were men. The mean aneurysm diameter varied between 47 and 75 mm. No studies reported the use of anticoagulants or antiplatelet agents. In all the studies, the principal method for IIA embolization was coils. The coils used were interlocking detachable coils (VortX; Boston Scientific, Natick, MA, USA), Interlock (Boston Scientific, Marlborough, MA, USA), Nestor (Cook Medical, Bloomington, IN, USA), Target embolism coils (Stryker Neurovascular, Fremont, CA, USA), detachable Concerto coils (ev3 Endovascular, Inc, Plymouth, MN, USA), magnetic resonance compatible platinum coils (Penumbra; Penumbra, Inc., Alameda, CA, USA), spirali (Pyramed) coils, and Concerto detachable coils (Medtronic, Minneapolis, MN, USA). The adjunctive method for embolization varied widely. The embolic liquid agents were Lipiodol (Guerbet Group, Roissy, France), Histoacryl (Tissue Seal, Ann Arbor, MN, USA), Onyx (Medtronic, Minneapolis, MN, USA), and n-butil cyanoacrylate (Histoacryl, Melsungen, Germany). Human thrombin was utilized in only one case. Amplatzer II vascular plugs were utilized in only one case (St. Jude Medical, Minnesota, MN, USA). The rate of technical success was 100%, suggesting that the embolic method is not crucial. For hemostasis percutaneous site access, coil embolization was utilized in two cases and closure device systems in two cases. Complications at the access site occurred in only one case, and coil embolization of the vessel was utilized. The follow-up length ranged from 3 months to 1 year in the present case. A comparative analysis of access vessels, techniques, and closure options utilized by different authors to treat CIA aneurysms caused by IIA retrograde flow is provided in Table 1.

## 4. Discussion

Late T2EL arising from IIA may occur after EVAR for aortoiliac aneurysm, with up to 16% occurring up to five years after the first procedure [1]. Nearly half of patients show aneurysmal sac growth and need intervention to avoid rupture. The pathogenesis of this condition is closely linked to the collateral pathway of the posterior trunk of the internal iliac artery. According to Werner Gibbins [5], secondary type II endoleaks in this setting are often the result of flow through patent iliolumbar or sacral artery collaterals, which are normally little branches hardly visible during conventional angiography due to the parallax effect and small caliber. In support of the thesis that T2EL in this setting comes from the iliolumbar or sacral arteries, a retrospective analysis of 53 endovascular iliac artery repair procedures by Heye [13], conducted to evaluate whether T2EL is more frequent in cases of antegrade residual flown versus total occlusion after coil embolization of IIA, demonstrated the same incidence of T2EL in both conditions and concluded that complete occlusion of the target vessel at the end of the procedure is not necessary to prevent type II endoleaks after endograft placement. In the same study, the authors pointed out that in all cases in which an endoleak was detected, a patent iliolumbar artery at the origin of the hypogastric artery was present, causing retrograde flow. That is the reason why several authors suggest identifying and occluding the iliolumbar artery when performing IIA embolization to avoid its retrograde flow with endoleak development during follow-up [14,15]. Other factors influencing endoleak appearance are the number and diameter of lumbar arteries, which are related to the aforementioned iliolumbar artery, and some authors have suggested their embolization in selected cases [16]. Alternative causes may be the hypertrophy of the vasa vasorum induced by the hypoxic environment in the arterial wall after covered stent deployment or inflammatory processes generated by the thrombus-derived contents, as explained by Patel and Fikani [4,17]. Despite their origin, T2EL from IIA true natural history is uncertain. They often resolve spontaneously, but in half of cases, endoleaks may persist, causing sac enlargement [1,18]. This occurrence may be a risky condition, and reported cases of iliac rupture during conservative management and poor results after emergency treatment suggest an elective approach [19]. Specific guidelines are absent, but there is agreement about treatment if the sac has expanded >1 cm over the past year or if a 5 mm expansion between two examinations is documented [20]. Past management techniques consisted of open surgery with ligation of the internal iliac artery, but less invasive procedures exist nowadays, so the current strategy reserves traditional surgery as a second option for suitable patients. The literature provides several treatment options suitable for different conditions, and a narrative review of relevant articles about type II endoleaks from IIA after EVAR was conducted to provide a summary of relevant treatment options. The pros and cons of each option were critically analyzed.

Aiming to reduce morbidity, laparoscopic aortic aneurysm repair has been proposed since 2001 [21]. Even reinterventions were conducted with the same technique, and a laparoscopic endoleak treatment was described by Zou et al. [22] in a particular case of type II endoleak of both the inferior mesenteric artery and the internal iliac artery, causing abdominal aneurysmal sac enlargement and pain. It allows the concomitant treatment of the two endoleaks with the same procedure in a relatively short time. However, the technical difficulty and the poor reproducibility of the procedure have limited its widespread use among vascular surgeons.

In recent years, the endovascular approach has advanced as the first choice due to its less invasive nature and efficacy. The endovascular goal is to interrupt the nidus flow by filling the nidus itself and embolizing feeding vessels. When direct hypogastric artery access is precluded by previous procedures, the endovascular route is challenging. In these cases, different ways are feasible. A direct anterior percutaneous trans-iliopsoas sac puncture has been described [23]. It has the potential benefit of directly reaching the nidus of the endoleak but requires no vital structures like bowel loops between the aneurysmal sac and puncture site. Because patient motion can influence the advancement of the needle, general anesthesia is required, but the major disadvantage is transferring the patient between the angio-suite and the CT scanner, which increases operative time as well as the risk of dislocation of the needle. Cone beam CT can reduce operative time, but it is not available in all angio-suites. A transosseous pelvic posterior CT-guided approach is described as an alternative to the anterior one when a safe route that prevents lesions of vital structures is not present, and it is chosen as the shortest, safest route into the aneurysm that avoids the bowel’s loop [24]. Possible complications of this technique include osteomyelitis, retroperitoneal hemorrhage, and fracture of the bone being transgressed. Considering the invasiveness of this procedure, it can be performed when no anterior ways are eligible and an absolute contraindication to the use of contrast iodine medium, like increased creatinine levels or allergy, has been shown by the patient. The transealing technique described by Coppi et al. [25] is a reliable option in which the nidus of the endoleak is reached through the virtual space between the arterial wall and represents a feasible and safe alternative in selected patients with T2EL suppling the aortic aneurismal sac. Unfortunately, it can only be applied safely when the graft lands on the common iliac artery. Otherwise, when the endograft lands on the external iliac artery, this technique is ineligible owing to the smaller caliber of the vessel and the risk of rupture or dissection. A different strategy can be used in chronic obstructive arterial disease, in which multiple collateral pathways that dilate with time are observed. The transcatheter arterial approach via the deep femoral artery collateral pathways linking to the internal iliac artery circulation has been used [26], but it is technically demanding due to the complexity of the collateral pathway itself if present. In our case, direct puncture of the aneurysm was avoided due to its anatomical features as well as the posterior approach for seronegative polyarthritis presented by the patient exposed to bone fracture. Therefore, we decided on a superior gluteal approach, which, in our opinion, is the shorter way to the main trunk of the internal iliac artery. Embolization through a percutaneous, retrograde, direct SGA access represents a simple way to reach the target vessel without entering the peritoneal cavity, crossing the iliac bone, or dealing with small collateral pathways. This approach has already been reported in recent years, and during the literature review process, we found nine papers that utilized the same method in 10 patients in total [5,6,7,8,9,10,11,12,16]. Direct surgical exposure or CT-guided puncture of the SGA could have been an alternative [10,27], but both fluoroscopic roadmapping and sonographic guidance provided a good visualization of the target vessel, allowing its puncture with a mini-invasive approach. In any case, multiple imaging supporting modalities are required, and CT guidance, DUS guidance, and fluoroscopic guidance alone or, most often, in combination are necessary. An innovative modality has been recently described by Chi et al., who utilized an 18-gauge (7 cm) SMART Doppler ultrasound-guided needle vascular access device (Vascular Solutions, Minneapolis, MN) to directly access the SGA [8].

Once the target has been reached, embolization of the aneurismal sac with or without embolization of feeding vessels is still debatable. It is not always possible to achieve the two targets, and procedures often end when one of the two has been realized. Several studies dealing with T2EL treatment focusing on the relative contribution of the two targets have failed to clarify whether sac embolization alone is better than sac occlusion associated with embolization of feeding vessels [13]. This aspect can be explained by the difficulty in revealing or reaching small collateral vessels or anatomic variants. After all, this review did not find evidence of any difference regarding short-term results in terms of sac enlargement, freedom from recurrence of EL, or reinterventions between the two techniques. Furthermore, the only case of post-procedural ischemic complication (buttock claudication) was observed after feeding vessel embolization (Table 1), and that is the reason why sac embolization may be deemed sufficient. The embolizing method, as shown by the results of the literature review, is not crucial, and coils alone or associated with embolic liquid agents can be used with excellent results.

At the end of the procedure, issues for access site hemostasis achievement arise due to the deep location of the SGA and the absence of bony structures to compress against. Hemostasis of the arteriotomy at the end of the procedure can be obtained with several techniques. Embolization of the access site has been proposed [12], but the potential risk is represented by distal ischemia. Alternatively, off-label use of arterial closure devices such as StarClose or Angio-Seal [8,11] can be utilized, but correct deployment of closure devices may be difficult when the target artery has a deep location and may require surgical exposure. Our strategy consisted of sheathless, low-profile devices that made the arteriotomy very small. Furthermore, heparin neutralization at the end of the procedure made manual compression successful. One aspect that was never considered in any of the studies examined in this specific setting of endoleaks arising from the internal iliac artery was the use of anticoagulants. However, there have been several studies comparing type II endoleak occurrence with abdominal aneurismal sac perfusion in patients treated with anticoagulation versus antiplatelet therapy alone. The first study postulating that anticoagulation can be an important factor influencing failure after endoluminal graft treatment was a case report by Torsello et al. [28], which reported a case of aneurysm rupture 16 months after a successful EVAR in a patient on coumadin due to atrial fibrillation. Even if no endoleak was detected at the scheduled CT scan, aneurismal sac enlargement was noted before rupture; after emergency treatment, the presence of a thrombus in the proximal neck and the concomitant anticoagulant therapy were recognized as possible factors for endograft failure. But the biggest study evaluating the long-term impact of anticoagulation on late endoleak occurrence after EVAR is that of Flohr et al. [29], who, over a retrospective cohort of 29,783 patients, found that late endoleaks were more common in patients treated with anticoagulation after EVAR.

## 5. Conclusions

The review of the literature suggests that direct SGA access represents a reliable route for treatment of T2EL arising from IIA when an anterograde access is not achievable. Embolization technique and access site hemostasis remain questionable. This report confirms that sac embolization through percutaneous SGA access and manual compression hemostasis is simple, safe, and effective, avoiding complications related to other access methods. However, all studies in this specific setting reported short-term results, and the longest follow-up was reported in our case with a 1-year CT scan.

## 6. Study Limitations and Future Directions

The present case discussion about the pathogenesis of type II endoleaks from the internal iliac artery reveals that it is still unclear. Efforts have to be made on preoperative imaging. The diagnosis of the precise location of endoleaks in these cases can be difficult with a CT scan alone, even if thin slices or late contrast phases are acquired, requiring, as shown in our case report, a diagnostic angiography to identify the exact origin. In fact, only after the angiography was it possible for us to plan a correct intraoperative strategy. If the diagnosis had been clarified before the therapeutic procedure, it would have been possible to better define the procedure. Therefore, our target as researchers is to identify these pathogenetic mechanisms to improve diagnosis. With this in mind, preoperative imaging needs to be as accurate as possible. Contrast-enhanced ultrasound (CEUS) is a non-invasive method largely used for the diagnosis of vascular pathologies in several districts. In fact, recent studies have demonstrated its beneficial contribution in carotid plaque characterization [30], in the follow-up of patients post-EVAR, and at the end of the EVAR procedures for the final angiographic control to verify the patency of the implanted endoprosthesis modules and the absence of type I endoleaks amendable to immediate treatment [31]. Unlike CT, CEUS allows visualization of the blood flow direction, which is an important tool to plan treatment, as in our case. However, CEUS is not yet a standardized method for the diagnosis of type II endoleaks from the iliac region. In our opinion, CEUS can be performed in patients with residual aneurysmal sac growth before surgery to help identify the origin of leaks.

## Figures and Tables

**Table 1:** Technical details of reported cases for endovascular treatment of Type 2 endoleaks.

Author, Year	SGA Access	Embolization Technique	Hemostasis	Complication	Follow-Up
Patel, 2023 [4]	DUS guided, 4Fr sheath 18G needle	Sac and feeding vessels embolization with microcoils	Manual compression	None	NA
Werner-Gibbings, 2013 [5]	CT guided, 17G needle, sheathless	Feeding vessels embolization with coils and sac embolization with liquid embolic agent	Manual compression	None	Not specified, stable sac
Herskowitz, 2014 [6]	Fluoroscopic + DUS guided, 21 G needle, 5Fr sheath	Feeding vessels embolization with coils + sac embolization with coils and thrombin	Embolization of the SGA with coil + manual compression	None	6-month CT scan, sac regression
Parlani, 2016 [7]	CT guided, 21 G needle, sheathless	Sac embolization with coils	Manual compression	None	3-month CT scan, stable sac
	CT guided, 21 G needle, sheathless	Sac embolization with coils	Manual compression	None	3-month CT scan, stable sac
Menon, 2018 [8]	DUS guided, Not specified	Feeding vessels embolization with coils	Angio-Seal	None	1-month CT scan, stable sac
Chi, 2018 [9]	Fluoroscopic + DUS guided, 18 G needle, 5Fr sheath	Sac embolization with coils and glue	Manual compression	None	CT scan, stable sac
Kim, 2021 [10]	CT + DUS guided, 21G needle, 3Fr sheath	Feeding vessels embolization with coils + sac embolization with coils and liquid embolic agent	Manual compression	Thigh and buttock mild claudication	1-month CT scan, stable sac
Norris, 2021 [11]	Fluoroscopic guided, 22G needle, 6Fr sheath	Sac embolization with coils, a liquid embolic agent, and a plug	StarClose	None	6-month CT scan, stable sac
Fukumoto, 2023 [12]	Percutaneous, DUS guided, 18G needle, 17G happycath	Feeding vessels + sac embolization with coils	Embolization of the SGA with coils	None	6-month MRI, stable sac
Present case	Angiographic + DUS guided, 18G needle, sheathless	Sac embolization with coils and a liquid embolic agent	Manual compression	None	12-month CT scan, stable sac

Legend: MRI, magnetic resonance imaging.

## Supplementary Materials

The following supporting information can be downloaded at: https://www.mdpi.com/article/10.3390/jcm13102909/s1, Video S1: Diagnostic angiography; Video S2: Sheatless SGA access.
